# Malignant melanoma: an audit on delay in diagnosis

**DOI:** 10.1186/1753-6561-9-S1-A20

**Published:** 2015-01-14

**Authors:** CWC Lee, FF Almutairi

**Affiliations:** 1 Department of Dermatology, Beaumont Hospital, Dublin, Ireland

## Background

A total of 67 patients diagnosed with melanoma at Beaumont hospital were included in this study. Our first aim was to investigate the effect of patient delay in seeking medical attention for any new or changing lesions on the Breslow thickness of the lesion and to explore factors that may have contributed to their delay. Secondly, we aimed to investigate the time interval between GP referral and dermatologist consultation and whether or not it is in accordance with the British Association of Dermatologist guidelines of two weeks.

## Methods

Patients diagnosed with malignant melanoma from 2009-January 2013 were identified using the dermatology biopsy logbook. Moreover, patient charts and a computerized database known as WINPATH were used to obtain our data. During our investigations, we were able to obtain 7 clinically relevant dates for our study. All of which had a potential for delay.

## Results

On average patients took 684 days (median 180) to seek medical attention after noticing a new or changing lesion. Furthermore, after patients attended their first medical consultation with their GP, they were examined by a dermatologist on average within 40.7 days (median 33) of referral. 94.1% (16/17) of patients who sought medical attention within 3 months presented with a lower Breslow thickness of < 1.5mm, while 52.6% (10/19) of patients who waited > 12 months had a higher Breslow thickness of > 1.5mm. 57.1% (8/14) of patients seen by a dermatologist within 2 weeks of their GP referral, presented with lesions <1.5 cm. While only 33.3% (14/42) of patients seen more than 2 weeks later, had a lesion greater than 1.5mm.

## Conclusions

In conclusion, patients included in our study waited on average 1.9 years before consulting their GP with a suspicious lesion. In addition, we managed to find a relationship between patient delay and the Breslow thickness of their melanoma. Furthermore, only 25% of the patients included in our study were seen by a dermatologist at Beaumont Hospital within 2 weeks of their GP referral. However, due to the limitations of our study we recommend that more extensive research be carried out on this issue.

